# PM_2.5_ Forecast in Korea using the Long Short-Term Memory (LSTM) Model

**DOI:** 10.1007/s13143-022-00293-2

**Published:** 2022-09-19

**Authors:** Chang-Hoi Ho, Ingyu Park, Jinwon Kim, Jae-Bum Lee

**Affiliations:** 1 School of Earth and Environmental Sciences, Seoul National University, 1 Gwanak-ro, Gwanak-gu, Seoul, 08826 Republic of Korea; 2 National Institute of Meteorological Sciences, Seogwipo, Republic of Korea; 3 National Institute of Environmental Research, Incheon, Republic of Korea

**Keywords:** AirKorea, Air pollution, Artificial intelligence, Community multiscale air quality (CMAQ), Long short-term memory (LSTM), PM_2.5_

## Abstract

The National Institute of Environmental Research, under the Ministry of Environment of Korea, provides two-day forecasts, through AirKorea, of the concentration of particulate matter with diameters of ≤ 2.5 μm (PM_2.5_) in terms of four grades (low, moderate, high, and very high) over 19 districts nationwide. Particulate grades are subjectively designated by human forecasters based on forecast results from the Community Multiscale Air Quality (CMAQ) and artificial intelligence (AI) models in conjunction with weather patterns. This study evaluates forecasts from the long short-term memory (LSTM) algorithm relative to those from CMAQ-solely and AirKorea using observations from 2019. The skills of the one-day PM_2.5_ forecasts over the 19 districts were 39–70% for CMAQ, 72﻿–79% for LSTM, and 73﻿–80% for AirKorea; the AI forecasts showed comparable skills to the human forecasters at AirKorea. The one-day forecast skill levels of high and very high PM_2.5_ pollution grades are 31﻿–98%, 31﻿–74%, and 39﻿–81% for the CMAQ-solely, the LSTM, and the AirKorea forecasts, respectively. Despite good skills for forecasting the high and very high events, CMAQ-solely forecasts also generate substantially higher false alarm rates (up to 86%) than the LSTM and AirKorea forecasts (up to 58%). Hence, applying only the LSTM model to the CMAQ forecasts can yield reasonable forecast skill levels comparable to the operational AirKorea forecasts that elaborately combine the CMAQ model, AI models, and human forecasters. The present results suggest that applications of appropriate AI models can greatly enhance PM_2.5_ forecast skills for Korea in a more objective way.

**Supplementary Information:**

The online version contains supplementary material available at 10.1007/s13143-022-00293-2.

## Introduction

As societies have become more technologically advanced, energy demands have increased to result in deterioration of air quality (AQ). To meet the clean-air mandate, the Korean government has implemented numerous policies to reduce the emission of air pollutants since the early 1980s (Kim and lee 2018; Trnka 2020). While the decrease of air pollutants concentration has been negligible since 2015 (Lee et al. 2021), the concentration of particulate matter (PM) with diameters ≤ 2.5 μm (PM_2.5_) was found to have decreased significantly from the 2019 winter to the 2021 (NIER 2021; posts dated January 10, 2022, in the press release on http://eng.me.go.kr/). The large reduction in the PM_2.5_ was most likely due to the social distancing precautions that were taken during the coronavirus disease (COVID-19) pandemic period; when COVID-19 protocols subsided, PM_2.5_ returned to the previous concentrations (Ju et al. 2021; Kwak et al. 2021). This is a great concern for the public health in Korea which has suffered from the phenomenon ‘*Sam-Han-Sa-Mi*’, which translates to the alternation between three cold and four polluted days.

The atmospheric PM concentrations are determined by multiple factors such as pollutants emissions, transboundary PM transports, ventilation, and scavenging (e.g., Choi et al. 2008, 2019; Lee et al. 2011; Oh et al. 2020; Chang et al. 2021). Unless there are certain events or strict reduction measures that reduce pollutant emissions, PM_2.5_ concentrations are expected to remain at elevated concentrations in the future. The National Institute of Environmental Research (NIER), under the Ministry of Environment of Korea, has been forecasting PM_2.5_ levels in four grades (low, moderate, high, and very high) over 19 districts nationwide since February 2 of 2015 through a national AQ forecast system, AirKorea (www.airkorea.or.kr) to help people to prepare for deteriorating AQ. The AirKorea system was organized with numerical modeling of Weather Research and Forecasting-Community Multiscale Air Quality (WRF-CMAQ) and subjective decision making by forecasters (Chang et al. 2016). Forecasters provide the PM_2.5_ grade forecasts for two-day lead-times based on the current weather conditions, the movement of air pollutants, and the CMAQ results. While several different types of artificial intelligence (AI) algorithms were introduced to improve the PM forecasting, results of these attempts were known to be challenging to interpret (c.f., Kim et al. 2022). Therefore, the use of the AI model requires experts with domain knowledge instead of its excessive trust (Zhang et al. 2021; Tjoa and Guan 2021).

In recent years, the application of AI in PM forecasts via including nonlinearity has significantly improved prediction accuracy (Wu and Lin 2019; Xayasouk et al. 2020). Incorporation of the weather and AQ variables from both observations and model forecasts into the AI-model training could reduce forecast errors considerably (Ho et al. 2021). While the long short-term memory (LSTM) algorithm is similar to a recurrent neural network (RNN) in that it learns a relationship among sequentially connected information, it compensates for the shortcomings of RNN by having a smaller effect on long-distance information (Hochreiter and Schmidhuber 1997; Gers et al. 2000).

The current LSTM-based PM_2.5_ forecast model is an improved version of the previous RNN model developed by Ho et al. (2021). The LSTM model modulates the CMAQ forecasts by incorporating an AI learning algorithm. The forecast skill of the LSTM model was compared to the AirKorea and CMAQ-solely forecasts. It is noted that the present CMAQ is slightly different from AirKorea’s; the configurations of the two models are described in supplementary Table S1. Section 2 describes the data and operational 19 forecast districts, and Section 3 explains the organization of the LSTM model. Section 4 presents a comparison of the prediction results of the LSTM, AirKorea, and CMAQ models. Section 5 summarizes the results of this study.

## Data and Forecast Districts

The inputs for the LSTM model were obtained from both observations and two model simulations (CMAQ and WRF), as described in Sections 2.1 and 2.2, respectively.

### Observed AQ and Meteorological Variables

**Fig. 1:**
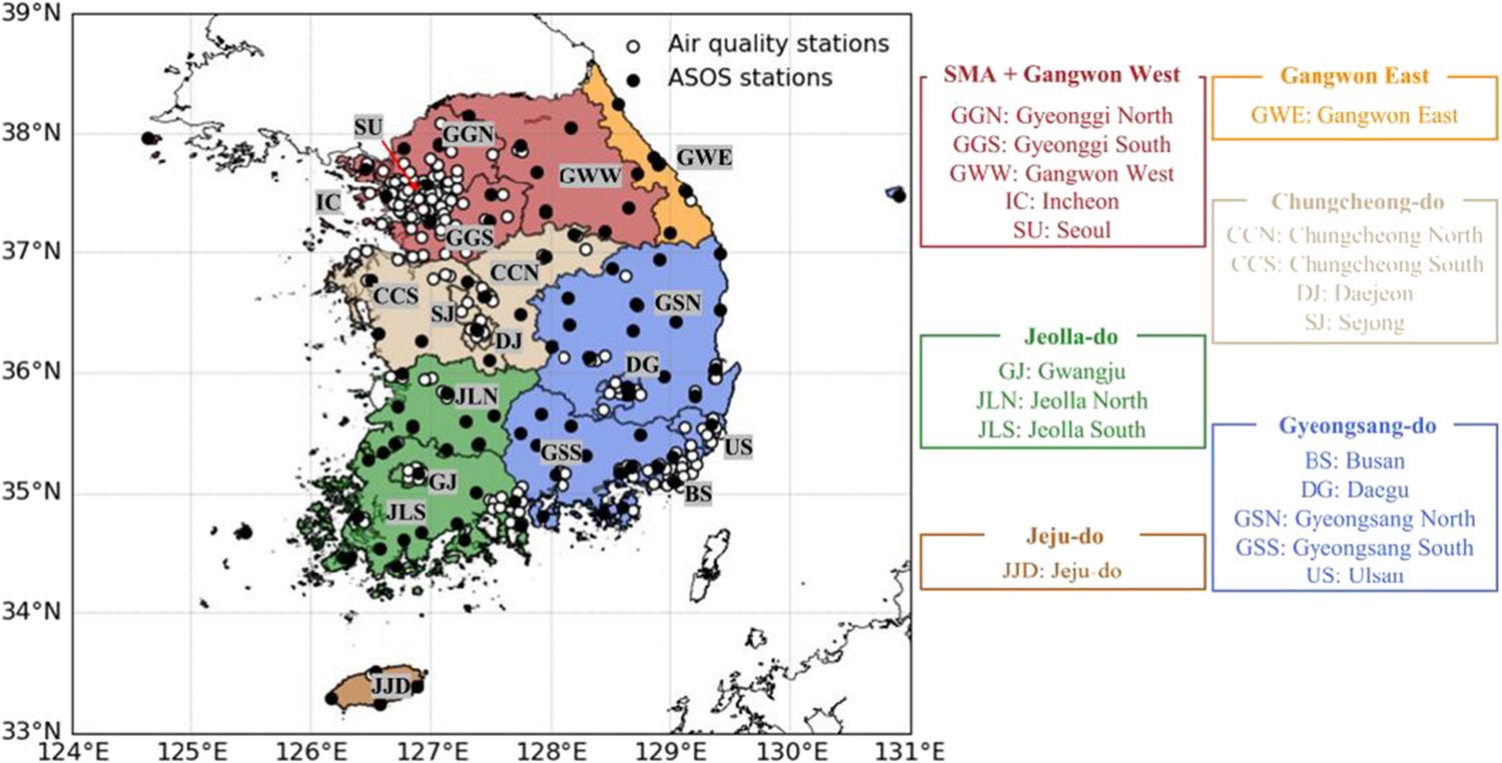
The location of air quality and weather stations in Korea. The 19 forecast districts are marked along with major cities and administrative provinces. The six broad regions are divided into different colors. The abbreviations of cities and provinces are shown in the right-side table

There are 260 AQ stations across Korea, mostly in and around megacities (denoted by open circles in Fig. 1). While Seoul is covered quite evenly by 25 stations in all administrative districts, there are often no observed stations in mountainous and/or rural regions, despite they cover substantially larger area than Seoul. Various 6-h mean values of AQ variables—PM of diameters ≤ 10 μm (PM_10_), PM_2.5_, O_3_, SO_2_, NO_2_, and CO—were analyzed for the period 2015﻿–21 (seven years; Table 1). Six-hour average meteorological variables (pressure, temperature, dew point temperature, relative humidity, and horizontal wind) at 97 automated surface observing systems (ASOS) across the nation (closed circles in Fig. 1) were also analyzed during the same seven years (Table 1).

**Table 1:** Air quality and meteorological variables used for input data to the long short-term memory (LSTM) model. Variables were obtained from both observations and model forecasts

	Types	Variables	Vertical levels
observations	air quality	PM_10_, PM_2.5_, O_3_, SO_2_, NO_2_, and CO	surface
	meteorology	pressure, temperature, dew point temperature, relative humidity, zonal and meridional wind	surface
model forecasts	air quality	PM_10_, PM_2.5_, O_3_, SO_2_, NO_2_, and CO	surface
	meteorology	geopotential height, temperature, relative humidity, zonal and meridional wind, and vertical wind	surface, 1000, 925, 850, 700, 500, and 300 hPa
	cosine similarity		1000, 925, 850, 700, 500, and 300 hPa
	back-trajectory cluster	probability of belonging to five back-trajectory clustering	500 m above the mean sea level

### Model Forecasted Results

The numerical AQ forecasts employ the CMAQ and WRF models for the simulations of AQ and meteorological data for driving CMAQ, respectively. There are four categories of the model results (Table 1). The first category includes the six CMAQ variables—PM_10_, PM_2.5_, O_3_, SO_2_, NO_2_, and CO—that were observed. The second is the WRF meteorological variables (geopotential height, temperature, relative humidity, and zonal, meridional, and vertical wind) at the surface and six atmospheric levels (1000, 925, 850, 700, 500, and 300 hPa). Third, cosine similarity and back-trajectory cluster values ​​are calculated by processing meteorological variables from the WRF. The cosine similarity is an index representing the spatial similarity to the meteorological fields for observed high PM_2.5_ concentration events (Hur et al. 2016), which are the same as the WRF meteorological variables at the same atmospheric level. Fourth, the back-trajectory cluster values are obtained by backtracking the air flow from the FLEXible PARTicle dispersion model (FLEXPART, Stohl et al. 2005; see http://flexpart.eu for details). FLEXPART has been widely utilized to identify the pathway of long trails of air pollutants. In this study, the air current flowing into the target district was tracked over the previous three days and the direction was indexed.

### Operational Forecast for Four Grades in 19 Forecast Districts

The CMAQ model forecasts PM_2.5_ concentration values at all grids. However, NIER produces AQ forecasts over the 19 districts in terms of four AQ grades. The four grades in the NIER operational forecasts are defined in terms of the PM_2.5_, as follows: low (PM_2.5_ ≤ 15 μg m^−3^), moderate (15 μg m^−3^ < PM_2.5_ ≤ 35 μg m^−3^), high (35 μg m^−3^ < PM_2.5_ ≤ 75 μg m^−3^), and very high (75 μg m^−3^ < PM_2.5_). The 19 districts were determined by considering the spatial distribution of AQ stations, populations, administrative districts, and consistency of living areas (Fig. 1). Eight big cities (Busan, Daegu, Daejeon, Gwangju, Incheon, Sejong, Seoul, and Ulsan) are regarded as independent districts. Sejong was selected as the second administrative capital, although its current population is around 380,889 (July 2022). Gyeonggi-do and Gangwon-do were divided into four districts: Gyeonggi North and South and Gangwon East and West, while each of the seven provinces (Chungcheong North and South, Gyeongsang North and South, Jeju Island, and Jeolla North and South) were designated as independent districts. Note that Jeju Island (*do* in Korean) is a special autonomous province, and is therefore denoted as Jeju-do.

Although the 19 districts are operationally independent, air pollutant concentrations varied similarly in geographically adjacent districts. For example, the daily anomalous PM_2.5_ concentrations in the Gangwon West and Seoul metropolitan area (including Seoul, Incheon, and Gyeonggi North and South; SMA hereafter) are strongly correlated with each other, with correlation coefficients of over 0.7 for the seven-year analysis period (significant at the 99% confidence level). The 19 forecast districts were therefore rearranged into six broad regions based on the interregional correlation coefficients when establishing the AI forecast model (table not shown). The six broad forecast regions colored differently in Fig. 1 are Chungcheong-do, Gangwon East, Gyeongsang-do, Jeju-do, Jeolla-do, and SMA + Gangwon West.

## Structure of LSTM Algorithm and Evaluation Metrics

### Flow Chart of LSTM Model Process

**Fig. 2:**
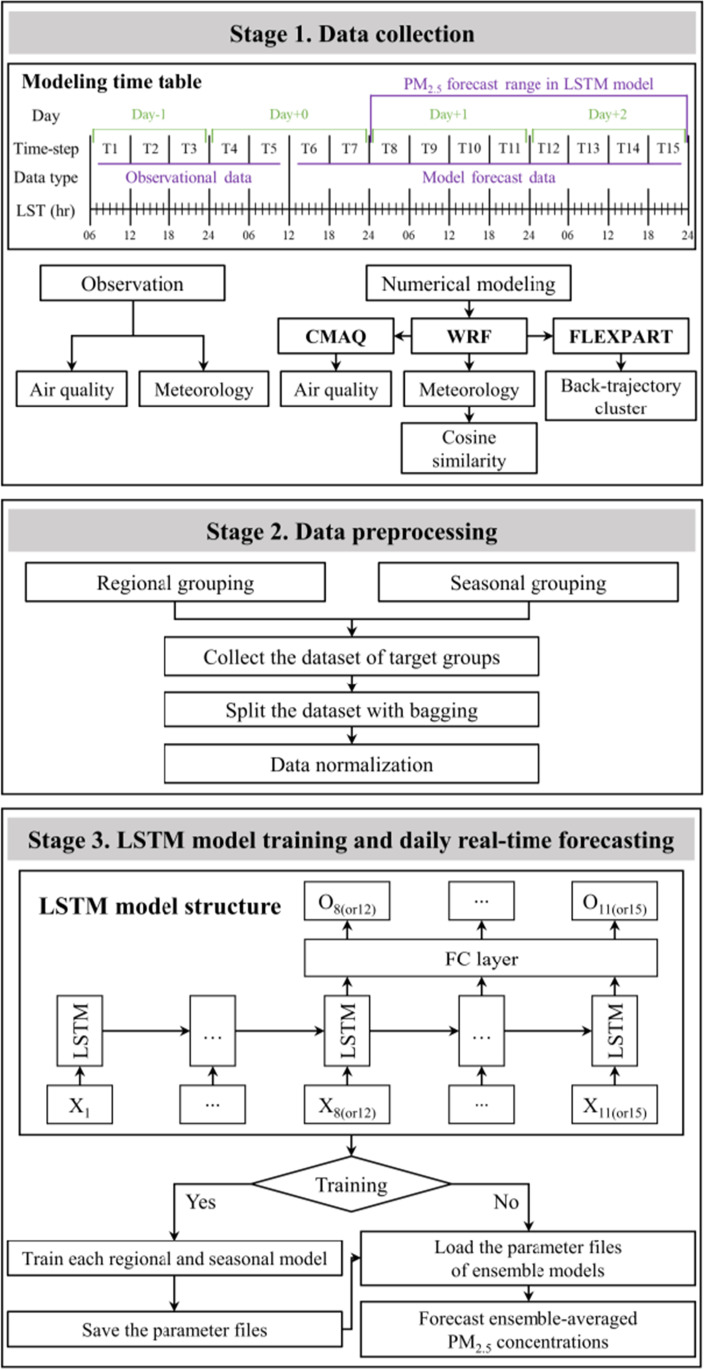
Three major stages in the present LSTM model: data collection, data preprocessing, and model training and forecasting

Figure 2 shows the three main components of the LSTM model: data collection, preprocessing, and model training/forecasting. Stage 1 (data collection) explains the sequence of the model time steps and the input data collection procedure. Each model run covered an 84-h period at 6-h time steps, from T1 (09:00 local time (LT) on Day−1) to T15 (21:00 LT on Day+2) in Fig. 2. The prediction time was at T5, which corresponds to 09:00 LT on Day0. The day forecast (Day0) was from T6﻿–T7. T1﻿–T3 corresponds to one day before Day0 (Day−1), T8﻿–T11 to one day ahead (Day+1), and T12﻿–T15 to two days ahead (Day+2). As explained in Sects. 2.1 and 2.2, AQ and meteorological variables used for LSTM input data were obtained from observations and model simulations.

In Stage 2, data preprocessing consisted of regional and seasonal grouping, bagging ensembles, and normalization. Operational forecasts are produced for the 19 districts and thereby for the six broad forecast regions comprised of neighboring districts (see Section 2.3). The primary AQ and weather variables in controlling PM_2.5_ concentrations varied seasonally. For example, a higher temperature and weaker ventilation occurred during stagnant periods, resulting in the deterioration of AQ. This implied that temperature and PM_2.5_ concentrations tended to be positively correlated, mostly in winter (Lee et al. 2021). However, there was a weak positive correlation between temperature and PMs in the summer. To consider the temporal variations in the influencing variables, the LSTM model was trained for 12 groups of three consecutive months (i.e., January-February-March, February-March-April, …, and December-January-February), as conducted by Ho et al. (2021). The bootstrap aggregating (i.e., bagging) ensemble method was as follows: N learners were generated through N random samplings from a dataset excluding a test set, which thereby averaged the predicted N outputs (Breiman 1996). An aggregation of multiple learners complemented the weaknesses of individual models, which resulted in higher performance. Our LSTM model had 40 learners for each regional and seasonal model, resulting in a total of 2880 (= 40 × 6 × 12) models. The testing year was 2019, and the remaining years (2015–18, 20, and 21) were arbitrarily separated into training (80%) and validation (20%) periods. For testing and validation, all datasets were normalized in the range of 0–1 using the minimum and maximum values of the training set.

Stage 3 depicts the training and operational forecasting processes of the model. For example, to forecast the PM_2.5_ concentrations in January in Seoul, 120 suitable models were selected from a total of 2880 models. In other words, 40 bagged ensembles for the November-December-January, December-January-February, and January-February-March models in the SMA + Gangwon West broad region were selected. Although our LSTM-based forecast system consisted of a large number of model sets, it was designed to enable a training process within 48 h (a few hours with two NVIDIA GeForce RTX 2080 Ti graphic cards). The detailed training procedures for the regional and seasonal models are described in the Appendix.

### LSTM Algorithm

The RNN algorithm was designed to search for linkages among all the variables in a given time series. The crucial weakness of the RNN method is the vanishing gradient problem, where there is a rapid decrease in memory information during an increase in the time interval between the information and forecast time. LSTM was developed to solve this problem, and is represented by Eqs. 1–6 (Gers et al. 2000).

1 $${\mathrm{F}}_{t}=\upsigma ({\mathrm{X}}_{t}{\mathrm{W}}_{f}+{\mathrm{b}}_{f}+{\mathrm{H}}_{t-1}{\mathrm{W}}_{f}+{\mathrm{b}}_{f}),$$

2 $${\mathrm{I}}_{t}=\upsigma ({\mathrm{X}}_{t}{\mathrm{W}}_{i}+{\mathrm{b}}_{i}+{\mathrm{H}}_{t-1}{\mathrm{W}}_{i}+{\mathrm{b}}_{i}),$$

3 $${\mathrm{G}}_{t}=\mathrm{sin}({\mathrm{X}}_{t}{\mathrm{W}}_{g}+{\mathrm{b}}_{g}+{\mathrm{H}}_{t-1}{\mathrm{W}}_{g}+{\mathrm{b}}_{g}),$$

4 $${\mathrm{O}}_{t}=\upsigma ({\mathrm{X}}_{t}{\mathrm{W}}_{o}+{\mathrm{b}}_{o}+{\mathrm{H}}_{t-1}{\mathrm{W}}_{o}+{\mathrm{b}}_{o}),$$

5 $${\mathrm{C}}_{t}=({\mathrm{F}}_{t}\times {\mathrm{C}}_{t-1}+{\mathrm{I}}_{t}\times {\mathrm{G}}_{t}),$$

6 $${\mathrm{H}}_{t}={\mathrm{O}}_{t}\times \mathrm{sin}({\mathrm{C}}_{t}),$$

where I_*t*_, F_*t*_, G_*t*_, and O_*t*_ denote the input, forget, cell, and output gates at the *t*-th time, respectively; W_*i, f, g, o*_ and b_*i, f, g, o*_ denote the weights and biases for each gate, respectively; C_*t*_ and H_*t*_ denote the cell and hidden states at the *t*-th time, respectively; σ denotes a logistic sigmoid function, and sin denotes a sinusoidal function (Sitzmann et al. 2020). In this study, the sinusoidal function was used instead of the hyperbolic tangent function, which is the basic activation function in a traditional LSTM cell.

**Fig. 3:**
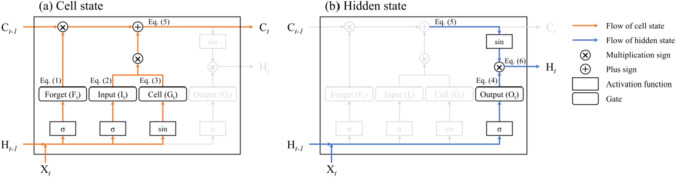
A flowchart of (**a**) cell state and (**b**) hidden state in LSTM memory cell structure. Orange and blue arrows describe the flow to find cell and hidden state at *t*-th time inside the LSTM cell, respectively. Rectangle boxes indicate either sigmoid and sinusoidal activation function and rounded rectangle boxes represent the internal gates (forget, input, cell, and output gates). The equation number is the same as in the manuscript

Figure 3 illustrates the flow of the cell and the hidden states inside the LSTM memory cell. Four gates (forget, input, cell, and output gates) played a role in adjusting state information. The cell state is a long-term memory device that encodes data over all time steps. The new cell state (C_*t*_) in the present time step was updated by combining the two processes of determining which information was loaded from the past and stored in the present (Fig. 3a). First, the forget gate (F_*t*_) removed trivial information about the cell state (C_*t-1*_) transferred from the previous time step (F_*t*_ × C_*t-1*_ in Eq. 5). Second, the input gate (I_*t*_) regulated the amount of information in the cell gate (G_*t*_) (I_*t*_ × G_*t*_ in Eq. 5). The output gate (O_*t*_) produced the necessary information from the activated cell state at each time step (Fig. 3b), which is called the hidden state (H_*t*_; Eq. 6). The hidden state is not the final output, but is encoded information that focuses more on the present time step, unlike the cell state. The internal process of the LSTM memory cell described above was performed for each time step.

### Evaluation Metrics

The CMAQ-solely, CMAQ-LSTM (LSTM hereafter), and AirKorea forecasts were evaluated against in situ observations. While observations, CMAQ, and LSTM provide PM_2.5_ mass concentrations (in μg m^−3^), the AirKorea forecasters designate AQ in four grades. For consistency with AirKorea forecasts, the PM_2.5_ mass concentrations values were converted into the four grades. The evaluation metrics included the root-mean-square error (RMSE), accuracy, probability of detection (POD), false alarm rate (FAR), receiver operating characteristic (ROC) curve, and area under the ROC curve (AUC). Because RMSE is calculated using the observed and model concentration differences (Eq. 7), it is not applicable to the evaluations of AirKorea forecasts. Accuracy represents the hit rate within the four grades (Eq. 8). POD denotes the rate at which the model detects both high and very high grades of public interest. As shown in Eq. 9, POD is the ratio of forecasts to observations for those two grades. The FAR is the rate of incorrect forecasts—for example, the high and very high grades against the observed low and moderate grades (Eq. 10).

The ROC curve represents the binary classification performance of alert (≥ a certain threshold) and non-alert (< a certain threshold) events, which are expressed in two axes: the true positive rate (TPR) on the y-axis versus the false positive rate (FPR) on the x-axis (Fawcett 2006). TPR is the probability of correctly forecasting the observed alert events (Eq. 11). The FPR is the probability of falsely rejecting non-alert events (Eq. 12). The alert (non-alert) refers to the days on which the forecasted or observed PM_2.5_ concentrations are above (below) the threshold value. If the threshold is 35 μg m^−3^, the alert is equal to high and very high grades, so POD and TPR display the same result. The AUC is a quantification of the ROC curve area (Eq. 13). As the point (FPR, TPR) approaches (1, 1) for any threshold in the ROC curve, the PM_2.5_ threshold becomes smaller. Therefore, most observations and forecasts become classified as alerts. On the other hand, as point (FPR, TPR) approaches (0, 0), the threshold becomes larger, which causes frequent non-alert events to occur in both observations and forecasts. Thus, if the curve is located on the upper left and the AUC is close to 1, the model shows a better performance.

7 $$\mathrm{RMSE}=\sqrt{\frac{1}{\mathrm{N}}\sum {(\mathrm{F}_{conc.}-\mathrm{O}_{conc.})}^{2}},$$

8 $$\mathrm{Accuracy}=\frac{\mathrm{F}_{low}\mathrm{O}_{low}+\mathrm{F}_{moderate}\mathrm{O}_{moderate}+\mathrm{F}_{high}\mathrm{O}_{high}+\mathrm{F}_{very \ high}\mathrm{O}_{very \ high}}{\mathrm{N}},$$

9 $$\mathrm{Probability \ of \ detection }\left(\mathrm{POD}\right)=\frac{\mathrm{F}_{high \ and \ very \ high} \mathrm{O}_{high \ and \ very \ high}}{\mathrm{O}_{high \ and \ very \ high}},$$

10 $$\mathrm{False \ alarm \ rate }\left(\mathrm{FAR}\right)=\frac{\mathrm{F}_{high \ and \ very \ high}\mathrm{O}_{low \ and \ moderate}}{\mathrm{F}_{high \ and \ very \ high}},$$

11 $$\mathrm{True \ positive \ rate }\left(\mathrm{TPR}\right)=\frac{\mathrm{F}_{alert}\mathrm{O}_{alert}}{\mathrm{O}_{alert}},$$

12 $$\mathrm{False \ positive \ rate } \left(\mathrm{FPR}\right)=\frac{\mathrm{F}_{alert}\mathrm{O}_{non-alert}}{\mathrm{O}_{non-alert}},$$

13 $$\mathrm{Area \ under \ the \ ROC \ curve }\left(\mathrm{AUC}\right)={\int }_{0}^{1}\mathrm{POD} \ d \left(\mathrm{FAR}\right),$$

where F_*conc.*_ and O_*conc.*_ denote forecasted and observed PM_2.5_ mass concentrations, respectively. N is the number of total samples, F and O with low, moderate, high, and very high subscripts respectively denote forecasted and observed PM_2.5_ grades, alert and non-alert subscripts are binary classifications divided by an arbitrary threshold, and F and O with numerator subscripts denote intersections (∩ in math) of forecast and observation, respectively.

## Evaluation of AQ Forecasts

The period of training, validation, and testing (i.e., evaluation) of the LSTM model should be chosen from among the seven years in which observations, CMAQ, and WRF simulations were prepared. The atmospheric PM_2.5_ concentrations reduced substantially in the two years of 2020 and 2021 due to the COVID-19 pandemic where the CMAQ and LSTM forecasts generally overestimated PM concentrations (not shown). For this reason, 2019, the pre-pandemic period, was taken as the evaluation period, and the remaining period including 2020﻿–2021 was used for the training and validation period. Although the inclusion of these two years in the training period strengthened the imbalance in the proportion for each grade, the overall performance was slightly improved in the LSTM forecast performance (not shown).

### Evaluation of the LSTM Forecasts

**Fig. 4:**
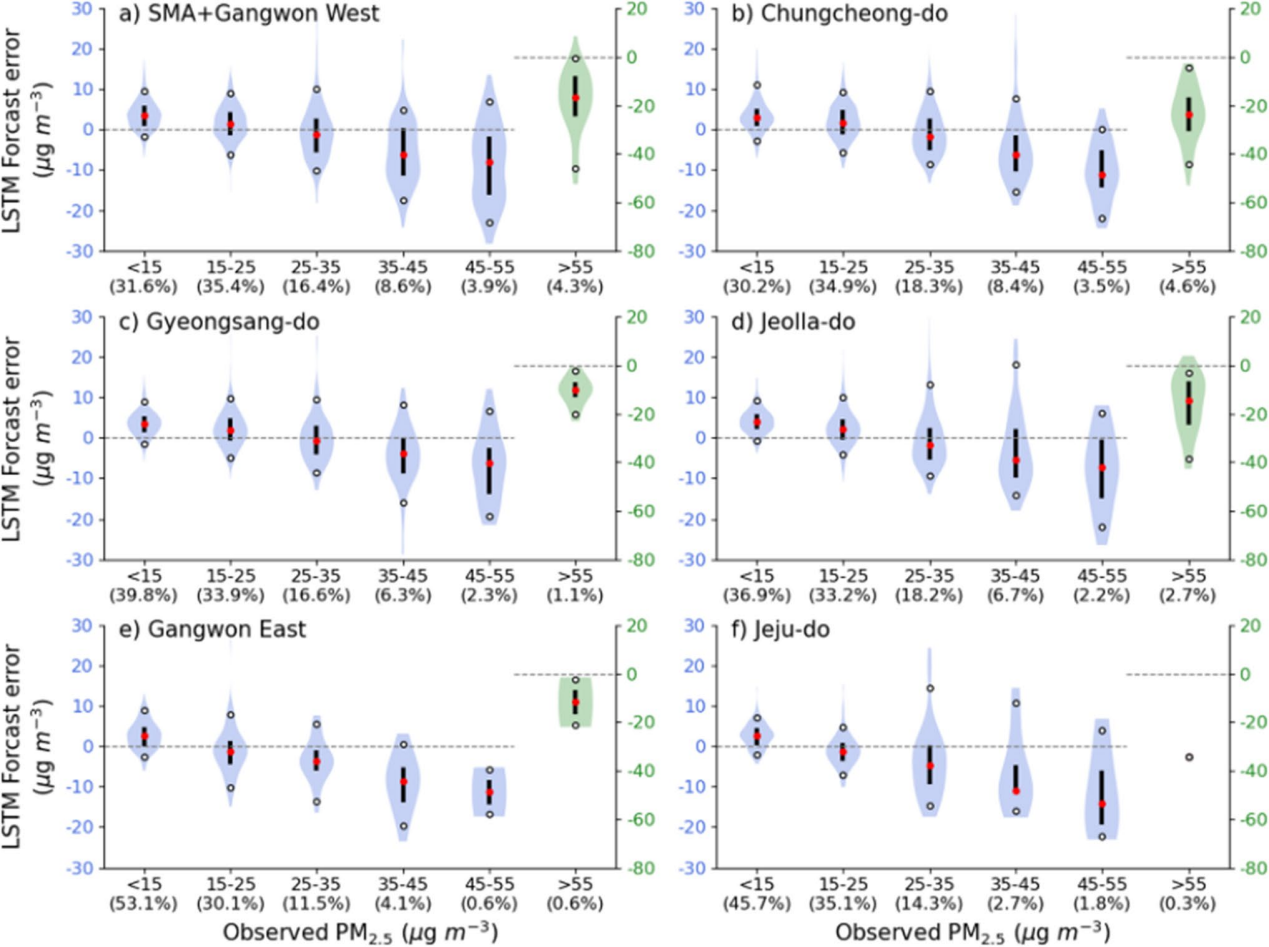
Violin plots of Day+1 LSTM forecast error according to observed PM_2.5_ concentrations in the six broad regions, (**a**) SMA + Gangwon West, (**b**) Chungcheong-do, (**c**) Gyeongsang-do, (**d**) Jeolla-do, (**e**) Gangwon East, and (**f**) Jeju-do. For > 55 μg m^−3^ bin, error values are displayed with different scale on the right y-axis. The number in parentheses on the x-axis is the percentage of the days included in the six PM_2.5_ bins

Figure 4 presents the errors in PM_2.5_ concentrations for Day+1 LSTM forecasts for the six broad regions. For convenience, the model errors were calculated and displayed at 10 μg m^−3^ intervals. Of the 365 days in 2019, the proportion of the days in each bin is shown in the parentheses beneath the x-axis. Depending on the region, the proportion of two bins < 25 μg m^−3^ is 65–83%, and that of 25–35 μg m^−3^ is 12﻿–18%; thus, the summed proportion in these three bins occupies over 83% of the entire year. The high and very high grades (bins ≥ 35 μg m^−3^) were most frequent (17%) in SMA + Gangwon West and Chungcheong-do (Fig. 4a and b) while the other four regions showed much smaller frequencies (< 12%) with the smallest in Jeju-do (Fig. 4c–f). The model forecast biases were positive/negative values for small/large PM_2.5_ concentrations below/above 25 μg m^−3^. The positive biases did not exceed 4 μg m^−3^ in the median value, but the negative ones were much larger, with a median value of −6 to −34 μg m^−3^ in three bins corresponding to the high and very high grades. It is supposed that the LSTM model underestimates high PM_2.5_ (≥ 35 μg m^−3^) concentrations. Despite these biases, the forecasted annual mean concentration agrees reasonably well with the observations.

The errors in Day+2 forecasts were similar to those of Day+1, except for larger absolute values (not shown). Note that the numerical weather prediction model errors increased with advancing forecast lead-time. In the LSTM forecasts, the effects of the model data were dominant over those of the observation data and contributed more to Day+2 than to Day+1 (Kim et al. 2022). The increase of errors in the WRF-CMAQ forecast with increasing forecast lead time amplified the LSTM model errors for longer the forecast lead times.

**Fig. 5:**
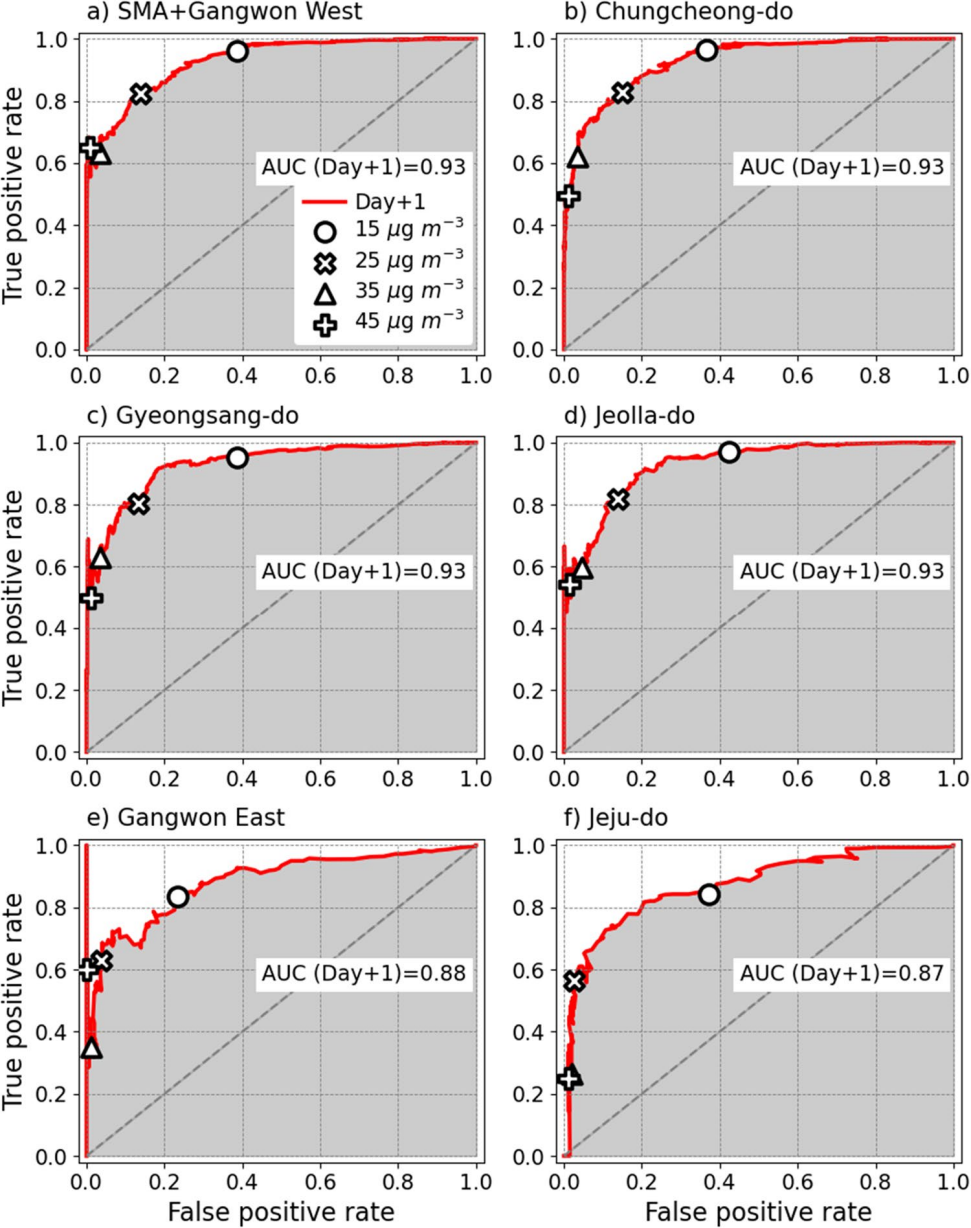
Distribution of the receiver operating characteristics (ROC) curves in the six broad regions, (**a**) SMA + Gangwon West, (**b**) Chungcheong-do, (**c**) Gyeongsang-do, (**d**) Jeolla-do, (**e**) Gangwon East, and (**f**) Jeju-do. Red line indicates ROC curve in Day+1 LSTM model forecasts. Four white-filled symbols (O, × , Δ, and +) show the ROC values at threshold (15, 25, 35, and 45 μg m^−3^) dividing alert and non-alert. The gray area represents AUC for Day+1

Figure 5 shows the ROC curve of the Day+1 forecasts for the six broad regions. The thresholds for the four points were the same as those in Fig. 4, except for 55 μg m^−3^. For the 15 μg m^−3^ threshold (O), the alert cases included the moderate, high, and very high grades, and the non-alert cases included the low grade. For the six broad regions, TPR ranged from 0.84 to 0.97, close to 1, but FPR was only 0.23﻿–0.42. The increase in FPR compared to TPR in the range of FPR ≥ 0.5 demonstrates that the PM_2.5_ concentrations were overestimated in the range < 15 μg m^−3^ as shown in Fig. 4. The 25 μg m^−3^ threshold (×) corresponded to the median of the moderate grade. At this reference point, the FPR and TPR values for four broad regions (SMA + Gangwon West, Chungcheong-do, Gyeongsang-do, and Jeolla-do) were 0.13﻿–0.15 and 0.80﻿–0.83, respectively, indicating satisfactory classification ability (Fig. 5a–d). At the 35 μg m^−3^ threshold (Δ), the FPR had a value close to 0 with little regional deviation. However, the TPR values were 0.26﻿–0.63, 0.17﻿–0.30 lower than those in the 25 μg m^−3^ threshold, showing a large regional discrepancy. In particular, in Gangwon East and Jeju-do, where PM concentrations were relatively low, the TPR was approximately 0.3, making the model vulnerable to more frequent high- and very high-grade alarms (Fig. 5e–f). For a threshold of ≥ 45 μg m^−3^ (+), the TPR decreased rapidly owing to the large negative bias in the LSTM model results (see Fig. 4). A few outliers were found in Gangwon East in the threshold range of 43–63 μg m^−3^ (Fig. 5e), though there were only four such cases (1.2%). The AUC of Day+1 was 0.87﻿–0.93, indicating that the LSTM model exhibited good forecast skill. Although not shown in the figure, the AUC of Day+2 was approximately the same as that of Day+1 in all broad regions.

The contributions of the input values to the LSTM model varied for each month, and the model performance varied from month to month. Table 2 illustrates the three evaluation parameters (accuracy, POD, and FAR) for Days +1 and +2 in the six broad regions for each season in terms of the four grades, not PM_2.5_ concentrations. The accuracy values remained generally similar across all six broad regions and were slightly larger (but statistically meaningless) in summer and fall than in winter and spring. The accuracy was 68﻿–85% on Day+1 and 2﻿–15% lower on Day+2. Second, PODs on Day+1 showed larger values compared to those of the annual (60%) and winter means (70%) in relatively more polluted regions (SMA + Gangwon West and Chungcheong-do). The values were 4﻿–16% smaller on Day+2. In contrast, PODs were below the annual mean of 39% in relatively more pristine regions (Gangwon East and Jeju-do), and below 56% in winter.

**Table 2:** The performance of LSTM forecast in Days +1 and +2. Numbers are in the order of annual/spring/summer/fall/winter. – denotes non-value

Broad region	Day	Accuracy (%)	POD (%)	FAR (%)
SMA + Gangwon West	Day+1	76 / 74 / 79 / 77 / 75	63 / 58 / 0 / 64 / 74	20 / 11 / – / 67 / 17
	Day+2	73 / 71 / 73 / 73 / 74	56 / 46 / 0 / 36 / 70	21 / 12 / – / 81 / 14
Chungcheong-do	Day+1	76 / 73 / 84 / 79 / 69	60 / 52 / 25 / 41 / 70	21 / 16 / 60 / 65 / 13
	Day+2	72 / 69 / 79 / 74 / 67	48 / 31 / 0 / 33 / 63	26 / 26 / 100 / 74 / 11
Gyeongsang-do	Day+1	77 / 76 / 78 / 77 / 76	61 / 35 / 62 / 17 / 76	32 / 44 / 27 / 50 / 30
	Day+2	72 / 73 / 74 / 69 / 75	45 / 11 / 27 / 17 / 65	35 / 56 / 36 / 80 / 29
Jella-do	Day+1	74 / 76 / 81 / 68 / 71	58 / 44 / 0 / 42 / 73	34 / 20 / 100 / 64 / 29
	Day+2	71 / 73 / 79 / 65 / 66	48 / 34 / 0 / 33 / 61	38 / 21 / 100 / 73 / 29
Gangwon East	Day+1	75 / 67 / 75 / 85 / 73	39 / 22 / – / – / 56	30 / 0 / – / – / 38
	Day+2	72 / 71 / 70 / 75 / 70	27 / 0 / – / – / 44	43 / – / – / – / 43
Jeju-do	Day+1	74 / 76 / 76 / 69 / 73	31 / 17 / – / 100 / 33	50 / 50 / – / 50 / 50
	Day+2	68 / 72 / 82 / 54 / 62	31 / 33 / – / 0 / 33	64 / 60 / – / – / 67

As FAR indicates the degree to which both high- and very high-grade forecasts fail, this value may increase with POD. Contrary to this expectation, however, the LSTM showed low FAR values for more polluted regions and seasons. The FARs were below 17% in regions SMA + Gangwon West and Chungcheong-do during winter, demonstrating LSTM’s excellent performance in forecasting both high and very high grades. The FAR values were somewhat greater in spring than in winter, and were even larger in summer and fall. In addition, the values were generally higher on Day+2 than on Day+1.

### Inter-Comparison of the Forecast Skill of the Three Models

**Fig. 6:**
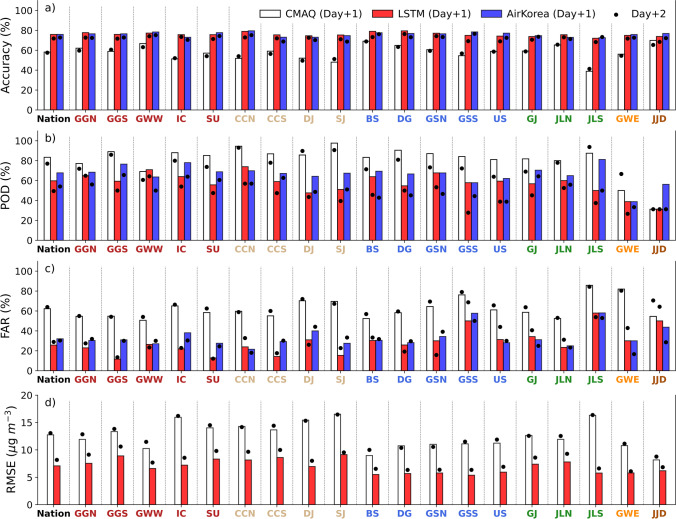
Comparison of the forecast skill of CMAQ-solely, LSTM, and AirKorea on Day+1 and Day+2 over the 19 districts, (**a**) accuracy, (**b**) probability of detection (POD), (**c**) false alarm rate (FAR), and (**d**) root mean square error (RMSE). The three models are displayed in different colors: white for CMAQ-solely, red LSTM, and blue AirKorea. The colored box shows values on Day+1 and the black dot shows values in Day+2

The performance of the CMAQ-solely, LSTM, and AirKorea forecasts for up to two days was evaluated for the 19 districts. Figure 6 shows the three forecast parameters and the RMSE for the forecast days and districts. The values on Day+1’s and Day+2’s are marked by colored boxes and black dots, respectively. It is apparent that the CMAQ-solely forecast exhibited low accuracy (~70%) with high POD and FAR values compared to the other two forecasts (Fig. 6a–c). Both high POD (31﻿–98%) and FAR (51﻿–86%) indicate that the CMAQ forecasts tended to overestimate PM_2.5_ concentrations (Fig. 6b and c; Ho et al. 2021). The national average values of accuracy and FAR on Day+2 were similar to those on Day+1, but the POD was 6% lower. The RMSEs of the CMAQ forecasts were 8.2﻿﻿–16.5 μg m^−3^ in both Days +1 and +2, indicating very large errors (Fig. 6d). Overall, the performance of the CMAQ-solely forecasts was demonstrated to be below optimal for operational purposes.

The LSTM and AirKorea forecasts showed similar forecast skill, too close to tell which one is better for operational purposes. For both LSTM and AirKorea, the accuracy was 68﻿–80%, POD was 27﻿–81%, and FAR was 12﻿–69% (Fig. 6a–c). LSTM showed a higher accuracy (~1.6%) than AirKorea for nine districts while AirKorea demonstrated higher accuracy for the remaining 10 districts (Fig. 6a). For most districts, the differences between the accuracies of Day+1 and Day+2 were less than 6%. The PODs of the AirKorea forecasts were approximately 8% above those of the LSTM forecasts, and 25﻿–31% higher for JLS and JJD than the LSTM forecasts (Fig. 6b). The LSTM forecasts yielded moderately smaller POD values (< 39%) for the GWE and JJD, where the CMAQ-solely forecasts produced noticeably low POD, 50% for GWE and 31% for JJD. The deterioration of POD (up to −﻿30%) Day+2 to Day+1 was greater than that of the accuracy. The AirKorea forecasts showed larger FAR than the LSTM forecasts, similarly as the POD from Day+1 and Day+2 (Fig. 6c).

The AirKorea forecasts were produced subjectively by the NIER forecasters based on the CMAQ forecasts and weather patterns, and given the analogous forecast skill of LSTM to that of AirKorea, it suggests that the forecast skill of LSTM is at the same level as that of trained human forecasters. The LSTM forecasts yielded an RMSE of 5﻿–9 μg m^−3^ on Day+1, about half of that in CMAQ forecasts (Fig. 6d). This value increased on Day+2 by 1.7 μg m^−3^ from Day+1.

**Fig. 7:**
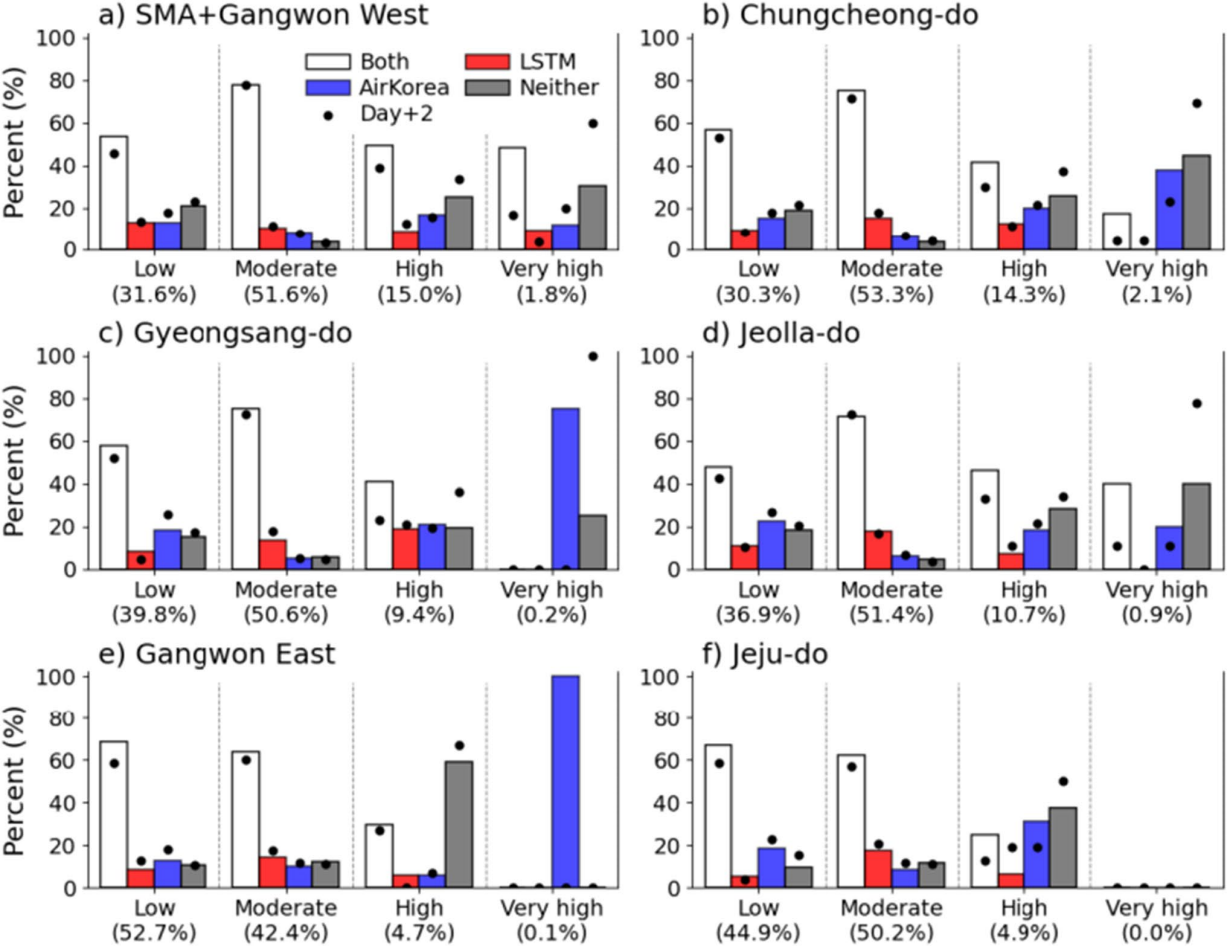
Accuracy of the grade forecast in Day+1 and Day+2 for LSTM and AirKorea over the six broad regions, (**a**) SMA + Gangwon West, (**b**) Chungcheong-do, (**c**) Gyeongsang-do, (**d**) Jeolla-do, (**e**) Gangwon East, and (**f**) Jeju-do. The colored box shows values in Day+1 and the black dot shows values in Day+2. The white bar (‘both’) means that both LSTM and AirKorea accurately forecasted the grade. The red (blue) bar indicates that LSTM (AirKorea) was correct and AirKorea (LSTM) was not correct, which is called ‘LSTM-solely’ (‘AirKorea-solely’) in the text. The gray bar (‘neither’) presents that both LSTM and AirKorea failed to forecast the grade

Figure 7 shows the accuracy of the LSTM and AirKorea forecasts across the four AQ grades in the six broad regions. In Fig. 6, the colored boxes and black dots denote the values on Day+1’s and Day+2’s, respectively. For the low and moderate grades, the two forecasts (‘both’ in the figure) demonstrated accuracies of 48﻿–78% across the broad regions. The hit rate for either the LSTM-solely’ or the ‘AirKorea-solely’ forecasts was ≤ 23%. The FAR in both (‘neither’ in the figure) was below 21%; the values for the moderate grade were even smaller. No significant differences in skill between Day+1’s and Day+2’s were found.

For the high and very high grades, the accuracy varied notably according to regions and forecasts, where ‘successes’ became smaller (mostly < 49%), and ‘no successes’ became larger (0﻿–60%). This implies that more efforts are needed to improve forecasting high PM_2.5_ events. When only one of the two forecasts was considered, the AirKorea-solely forecasts were better than the LSTM-solely forecasts for the very high grade, with success ratios of 38% for Chungcheong-do, 75% for Gyeongsang-do, and 100% for Gangwon East (Fig. 7b, c, and e, respectively). For Jeju-do, a very high grade event never occurred as correctly forecasted by both forecasts (Fig. 7f).

Table 3 compares the accuracy averaged over the 19 districts according to the grades and seasons for the LSTM and AirKorea forecasts. The accuracy was highest for the moderate grade in all seasons and for forecast lead times (i.e., Days +1 and +2). Both systems simultaneously failed (i.e., ‘neither’) for up to 10%. For the moderate grade, the percentages where both forecasts were accurate (i.e., ‘both’) were 75﻿–78% for Day+1 and 53﻿–58% for Day+2 except for fall. Accuracy of the LSTM forecasts (i.e., combining both and LSTM-solely) was 85﻿–93% on Day+1 and 84﻿–92% on Day+2, higher than that of the AirKorea-solely forecasts (75﻿–84% on Day+1 and 44﻿–63% on Day+2) on both Day+1 and Day+2.

**Table 3:** Accuracy (%) of the four-grade forecast in Day+1 and Day+2 for LSTM and AirKorea averaged over all 19 districts. Numbers are in the order of both/LSTM-solely/AirKorea-solely/neither. – denotes non-value. The meaning of ‘both’, ‘LSTM-solely’, ‘AirKorea-solely’, and ‘neither’ is the same as Fig. 7

Day	Season	Low (%)	Moderate (%)	High (%)	Very high (%)
Day+1	Spring	39 / 14 / 17 / 30	77 / 16 / 4 / 3	35 / 6 / 26 / 32	47 / 3 / 26 / 24
	Summer	68 / 6 / 14 / 12	78 / 11 / 6 / 5	13 / 17 / 15 / 55	–
	Autumn	62 / 9 / 18 / 11	65 / 20 / 10 / 5	26 / 19 / 23 / 32	–
	Winter	34 / 15 / 18 / 32	75 / 10 / 9 / 7	56 / 13 / 15 / 17	21 / 5 / 28 / 47
Day+2	Spring	6 / 42 / 10 / 42	58 / 35 / 5 / 3	6 / 20 / 14 / 60	0 / 16 / 0 / 84
	Summer	26 / 40 / 11 / 23	53 / 37 / 5 / 5	2 / 10 / 7 / 82	–
	Autumn	38 / 24 / 24 / 14	37 / 47 / 7 / 9	0 / 31 / 6 / 62	–
	Winter	18 / 27 / 23 / 33	53 / 32 / 9 / 5	11 / 50 / 10 / 29	0 / 12 / 2 / 86

For the low grade, ‘both’ decreased and ‘neither’ increased significantly. This change is obvious for Day+2, where they reached 6﻿–38% and 14﻿–42%, respectively. For Day+1, AirKorea-solely forecasts showed higher skill than the LSTM-solely forecasts; the opposite was true for Day+2. For the high and very high grades, both forecasts exhibited smaller values than those for the low grade. For the two highest grades, most forecasts of the two systems did not exceed 50% accuracy on Day+1. The accuracy ratio further worsened (up to 86% for neither) on Day+2.

## Conclusion

This study evaluated two-day PM_2.5_ forecasts for Korea using three forecast systems, CMAQ-solely, CMAQ-LSTM, and AirKorea over the seven-year period 2015–21 with the year 2019 as the testing (or evaluation) period; the remaining years were used as the training and validation periods. Including the anomalous years of 2020﻿–21 due to the COVID19 pandemic in the evaluation period yielded essentially identical results as presented in this study (not shown). The results for the six broad regions showed median LSTM forecast error and observation error values to be −4.6 and 4 μg m^−3^ for the low and moderate grades, and −34.1 to −4 μg m^−3^ for the high and very high grades. In the ROC curve that represents the classification performance of alert and non-alert divided by an arbitrary PM_2.5_ thresholds, the LSTM model yielded the best skill for the moderate grade with near-zero bias. For the threshold values of the low-grade, those of the TPR were nearly one, and the FPR was larger than 0.4. In contrast, for the high and very high grades, FAR was nearly zero, and the TPR decreased to below 0.6. The overall performance of the LSTM model was acceptable, with AUC over 0.87 and 0.84 in Day+1 and Day+2, respectively.

Averaged over the all broad forecast regions with the grade-based evaluation of the LSTM forecasts, the accuracy, POD, and FAR values on Day+1 (Day+2) were 76% (72%), 60% (50%), and 26% (29%), respectively. While the accuracy remained similar for all broad regions and seasons, POD exhibited high values in polluted regions (SMA + Gangwon west and Chungcheong-do) and during winter, and FAR showed high values in pristine regions (Gangwon East and Jeju-do) and during all seasons except for in winter. Furthermore, the LSTM forecasts were compared against the CMAQ-solely and AirKorea forecasts. The CMAQ forecasts underperformed the other two forecasts, with low accuracy and high FAR due to overestimation. The average RMSE of the CMAQ forecasts for Day+1 was 12.8 μg m^−3^, which is 1.8 times that of the LSTM model. AirKorea, on the other hand, showed similar accuracy as LSTM, yielding 8% higher POD and 6% higher FAR compared to those from the LSTM on Day+1. Both models showed high accuracy for low and moderate grades across all the broad regions. However, for high and very high grades, the hit ratios decreased to 49% and the failure ratio increased to 60% along with large regional deviations.

Using the LSTM-based AI model in conjunction with CMAQ-based AQ forecasts has yielded a performance level similar to experienced human forecasters who used subjective interpretation of observations and/or numerical model predictions. However, this does not warrant that the AI model can replace the human forecasters. The AI model has inherent limitations: a black box for which the decision-making processes in producing forecasts were not interpreted at all. It is also a major weakness that the cause of incorrect forecasts yielded by the AI is not explained. Although research on explainable AI actions has been actively conducted to solve this problem, it is currently insufficient. Moreover, the LSTM model was highly dependent on inputs from the numerical model forecasts. Because an advanced numerical model makes the AI model more complete, the development of numerical models as well as AI models is necessary. Therefore, current LSTM forecasts can only be recommended as additional references for human forecasters, something that the NIER has already begun to do.

### Electronic supplementary material

Below is the link to the electronic supplementary material.

Supplementary file1 (DOCX 18 KB)

## Figures and Tables

**Algorithm 1:**
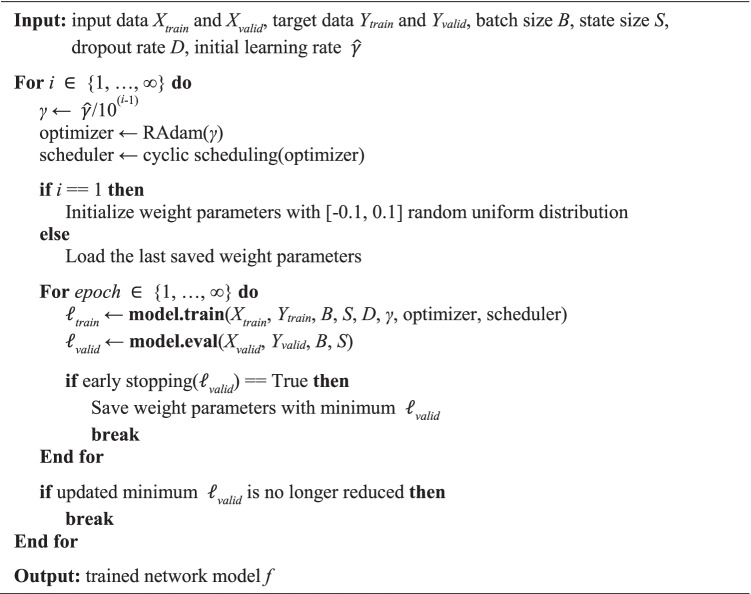
Algorithm: Training process of LSTM model

## Appendix

The training process of the LSTM model was applied identically to all the districts and seasons. For the training, two input variables (*X*_*train*_ and *X*_*valid*_) and two output variables (*Y*_*train*_ and *Y*_*valid*_) during the training/validation period and hyperparameter values (batch size *B* = 64, state size *S* = 10, dropout rate *D* = 0.1, and initial learning rate $\widehat{\gamma }$ = 0.001) needed to be assigned first. The rectified Adam (RAdam) optimizer and cyclic learning rate scheduling techniques were utilized (Liu et al. 2019; Smith 2017). The training process was terminated by using the early stopping method. In many cases, the training loss ($\ell$_*valid*_) value continued to decrease, but the validation loss ($\ell$_*valid*_) value increased after a certain epoch. This feature appeared when the model was overfitted. Early stopping is a method to halt the training process when the minimum value of $\ell$_*valid*_ is no longer renewed. The optimal model was the weight parameter of the model with the minimum value of $\ell$_*valid*_ is stored. The LSTM algorithm was designed to repeat multiple training phases to optimize the model.

The initial values of all trainable weight parameters had a randomly uniform distribution in the range of (−0.1, 0.1) at the start of the training process (*i* = 1, the first training phase). Then, in the model training () at each epoch, the trainable parameters were adjusted to minimize $\ell$_*valid*_ value. The model eval () did not update the parameters but was used to determine whether the calculated $\ell$_*valid*_ values met the early stopping condition. When the first training phase terminated, the second training phase (*i* = 2) began immediately. After the second training phase, γ became smaller than $\widehat{\gamma }$, which was the process of searching for local minima in a narrow range by making parameter changes smaller. For consecutive training, the weight parameters of the model saved in the first training phase were used as the initial parameter values for the second training phase. In this manner, the training was repeated and terminated when the minimum validation loss (${\ell}_{valid}^{N}$) of the N^th^ training phase was no longer less than the minimum ${\ell}_{valid}^{N-1}$ of the N−1^th^ training phase.
