# Supplementary material for: PM2.5 Forecast in Korea using the Long Short-Term Memory (LSTM) Model
Source: Asia Pac J Atmos Sci. 2022 Sep 19:1–14. Online ahead of print. doi: 10.1007/s13143-022-00293-2 (PMC9483905; doi:10.1007/s13143-022-00293-2)
Supplement: Supplementary file 1 — Supplementary file1 (DOCX 18 KB) [file 13143_2022_293_MOESM1_ESM.docx]

Table S1. Configuration for the present and AirKorea’s numerical model system.

| Model | Option | | Our | AirKorea |
| --- | --- | --- | --- | --- |
| SMOKE | Version | | v2.7 | v3.1 |
|  | Emission | China | MEIC 2010 | Unknown |
|  |  | East Asia | REAS 2008 | MICS-Asia 2010 |
|  |  | Korea | CAPSS 2011 | CAPSS 2010 |
| CMAQ | Version | | v4.7.1 | v4.7.1 |
|  | Physics / Chemical scheme | Chemical mechanism | CB05 | SAPRC99 |
|  |  | Aerosol module | AERO5 | AERO5 |
|  |  | Chemistry solver | EBI | EBI |
|  |  | Horizontal advection | Yamo | Yamo |
|  |  | Horizontal diffusion | Multiscale | Multiscale |
|  |  | Vertical diffusion | Eddy | Eddy |
| WRF | Version | | v3.6.1 | v3.6.1 |
|  | Initial and boundary conditions | | GFS | UM |
|  | Physics scheme | Microphysical | WSM6 | WSM3 |
|  |  | Long wave radiation | RTTM | RTTM |
|  |  | Short wave radiation | Dudhia | Goddard |
|  |  | Planetary boundary layer | YSU | YSU |
|  |  | Cumulus | Kain-Fritsch | Kain-Fritsch |
